# Cases from the Surgical Clinic of Jefferson Medical College

**Published:** 1860-11

**Authors:** Emil Fischer

**Affiliations:** Philadelphia


### ART. II.—*Cases, with Remarks, from the Surgical Clinic of the Jefferson Medical College.*—Service of PROFESSOR GROSS.

Reported by EMIL FISCHER, M.D., of Philadelphia.

### I.—*Two Cases of Spina Bifida; Treatment by Injections of Iodine; Death.*

*Case* 1.—James Grove, a healthy infant, aged eleven weeks, was admitted into the Clinic of the Jefferson Medical College, in October, 1859, on account of a congenital tumor over the lumbar region. The swelling, which at the time of birth had been as large as a walnut, had gradually increased to its present size, which was that of a large orange. It was of globular form, narrow at its base, and was situated in the median line, over the spinous processes of the last lumbar vertebræ. The integuments covering it were much attenuated, particularly at the top of the tumor, where they seemed ready to burst; they were reddened by capillary injection, the discoloration being most intense at the lower part of the tumor and around its margin. The tumor was translucent, and distinctly fluctuating; compression did not decrease its bulk; no impulse was imparted to the finger, placed on the fontanel, when the tumor was pressed, nor did the pressure produce any pain or convulsions.

The mother stated that the child had been born at full term, and had always enjoyed good health; it had perfect use of both lower extremities, and its urine and feces were under the power of the will. The head was of natural size, and there was no club-foot, or other deformity. The skin over the tumor had been, at some previous time, the seat of superficial ulceration. Professor Gross pronounced the case to be one of spina bifida, and taking into consideration that pressure upon the tumor did not produce any effect upon the nervous centres, and that the functions of the body were not disturbed to any appreciable degree, he judged that the orifice, by which the contents of the tumor communicated with the spinal canal, was but small. The prognosis, unfavorable from the nature of the disease, was rendered still more so by the rapid increase of the tumor, and by the highly attenuated state of the integuments which seemed to be on the point of giving way. The parents of the child being anxious to have something done for the cure of the disease, and being willing to run the risk of an operation, Professor Gross decided to treat the case by injections of iodine, according to the method proposed, and practiced with such encouraging results by Professor Brainard, of Chicago.

On the nineteenth of October, a puncture was accordingly made in the sound skin, about one inch and a quarter from the base of the tumor, by means of a flat, curved needle, and the instrument having been carried subcutaneously into the tumor, one drachm of its serous contents was allowed to escape; a solution of one-eighth of a grain of iodine and one quarter of a grain of iodide of potassium in one drachm of water was then injected by means of a small syringe, provided with a delicate, curved nozzle. A dressing of adhesive straps and collodion was applied over the puncture, but being found insufficient to prevent leakage, the puncture was closed by the twisted suture. The tumor was thoroughly painted over with collodion. Three drops of tincture of opium were administered to the patient, and directions were given to keep him lying on the face.

On the second day after the operation a small opening was discovered at the top of the tumor, from which there was a slight drainage. The child had had some convulsions during the previous night. To prevent the escape of more fluid the tumor was covered with several successive layers of cotton, painted over with collodion, until a sheath of about one-eighth of an inch in thickness was formed.

On the morning of the next day the tumor appeared to be less tense. The child had passed a comparatively comfortable night, and took the breast well. A dose of castor-oil was ordered to keep its bowels soluble. On the evening of the same day an escape of fluid from the tumor was again noticed, and was checked by another application of cotton and collodion. The dressing was, however, inadequate to control the discharge permanently, and on the morning of the fourth day the tumor was found leaking again; this time the fluid escaped from the point where the twisted suture was applied. The child had suffered from convulsions during the previous night; it had a haggard appearance, and seemed much sunken. Cotton and collodion were applied as before.

On the fifth day after the operation, the tumor burst completely, all its contents escaping suddenly through a large rent at its top. The child died under severe convulsions.

The autopsy was made twenty-four hours after death. Only the tumor was allowed to be examined. On laying the sac completely open, its lining membrane was observed to be highly injected, and coated with a thin layer of lymph, which in some places was softened and broken down. The opening by which the cyst communicated with the spinal canal was but of small size; it consisted of a cleft, about a quarter of an inch wide, involving the arch of the fifth lumbar vertebra, the spinous process of which was wanting. No nerve-twigs could be discovered passing out from the spinal canal into the interior of the sac.

*Case* 2.—Charles Lehmann, three months of age, was brought to the Clinic of the Jefferson Medical College, in October, 1859, on account of a tumor over the lumbar region. The child's health was excellent, and it was perfectly well developed in every other respect. The tumor was situated in the median line over the lower portion of the lumbar region, projecting somewhat over the sacrum. It was globular, about as large as a medium-sized billiard ball, translucent, fluctuating, and elastic. The skin covering it was not discolored, but thin and shining, although by no means as attenuated as in the case reported above. Upon pressure, the size of the swelling was slightly diminished, but no fluctuation could be perceived at the fontanels. The child moved its lower extremities convulsively whenevever the tumor was touched with any degree of force. At the time of birth the tumor was flabby, soft, and hardly half of its present size. Its enlargement had taken place very gradually. The child had the perfect use of its limbs, and all the functions of the body were regularly performed. Considering the more advanced age of the child, the smaller size and slower growth of the tumor, and the more favorable condition of the integuments, this case seemed to be better suited to the treatment by iodine than the preceding, and Professor Gross, therefore, on the 19th of October, 1859, threw into the swelling, the puncture having been made as in the other case, a drachm of water, containing one-eighth of a grain of iodine and the fourth of a grain of iodide of potassium. The puncture had to be closed by the twisted suture, a very delicate cambric needle being used, and the whole tumor was thoroughly coated with collodion. As in the other case, the recumbent position on the abdomen was enjoined, and was carried out as well as possible.

In the evening after the operation the child had slight convulsions, which were relieved by three drops of laudanum. The operation was repeated on the twenty-sixth of October, and again on the second of November, by which time considerable diminution in the volume of the tumor had taken place. From the latter date to the twenty-third of December, when the case terminated by death, the injections were thrown in every fourth or fifth day, the quantity of iodine being increased to one-sixth of a grain. Collodion was applied twice a day, and just before the patient died the tumor had diminished two-thirds, leading Professor Gross to suppose that the case might soon terminate in a cure. The child rarely suffered from convulsions after the operations, and they always readily yielded to a dose of castor-oil. Whenever there was pain, a few drops of laudanum were administered, with a happy effect.

The child died in convulsions on the twenty-third of December, the tumor having burst and discharged its contents. An autopsy was made at the expiration of sixteen hours. The cavity of the sac was found to be about one-third obliterated by coagulated lymph. The fissure involved the posterior portion of the last lumbar vertebra and the first piece of the sacrum, but the arachnoid on each side of the fissure was covered with lymph, which caused the gap to be of smaller size than it was originally. Had the patient lived a few weeks longer, a cure might possibly have resulted.

*Remarks.*—On presenting these cases to his class, Professor Gross made the following remarks: The two cases which at present engage our attention exhibit a form of congenital malformation, known to pathologists by the name of *hydrorachitis*, or *spina bifida*. It consists of a fluid swelling, constituted by the membranes of the spinal cord, which are distended and protrude backward through a congenital cleft of the vertebral column.

This affection, which is closely analogous in its character to congenital hydrocephalus, with which it is frequently associated, is attended with an abnormal collection of fluid within the membranous sac of the spinal column, from which it has received the name of hydrorachitis, or dropsy of the spine. Whether this collection of fluid is owing to inflammation of the membranes of the spinal cord, disturbances in the nutrition of the part, or other causes operating during intra-uterine life, it is impossible to say. The most common seat of the serous effusion is the sub-arachnoidean space, the fluid of which is thus augmented beyond its normal quantity, and may amount from a few ounces to several pounds. In some exceedingly rare cases the exudation has been found to occupy a space between the dura mater and the bony walls of the vertebral column. The fluid in hydrorachitis is generally of a thin, limpid character, slightly saline in its taste, and almost uncoagulable. Sometimes, however, it is glutinous, yellowish, sanguinolent, or contains flakes of lymph and particles of pus, appearances which are attributable to secondary processes of an inflammatory character.

Although dropsy of the spine is accompanied, in the generality of cases, by more or less extensive malformation of the vertebral column, it may exist without any such malformation. In the latter case, the vertebral column remaining in a normal condition, the disease may not give rise to any outward deformity, or it may, as has been observed in some very rare instances, cause a sac, formed by the membranes of the spinal marrow, to protrude between two transverse processes from the canal, in the cervical or lumbar region, and to effect a lodgment immediately underneath the external integuments.

The malformation of the spine with which the affection is usually attended, and from which it has received the name of spina bifida, consists in a deficiency of the vertebral rings, owing to an arrest of ossification, and may be compared to those defects which originate from a want of union of the two halves of the fœtus during utero-gestation, such, for instance, as hare-lip and cleft palate. It may be arranged under the following heads: 1. Division of the entire vertebra, even of its body. 2. Partial or complete absence of the lateral arches. 3. Perfect development of the lateral arches with want of union at the median line. In the highest degree of spina bifida the anomaly involves the whole length of the vertebral column, and is then generally associated with extensive destruction of the spinal marrow. In the more common cases, however, such as are likely to come under the observation of the surgeon, the cleft in the spinal canal is but partial, affecting usually the lumbar region, occasionally, however, the dorsal, cervical, or even the sacral. The protrusion of the membranes of the spinal marrow through this cleft generally takes place during fœtal life; in some instances it does not occur until some weeks or months after birth. The tumor varies much in size, being sometimes so small as to be hardly perceptible, at other times attaining the size of a child's head; a variation which seems to depend, in a great measure, upon the smaller or greater extent of the cleft. Usually the swelling does not exceed the size of an orange. In its form it is globular, ovoidal, or pear-like, with a short and more or less constricted neck. The anatomical composition of the walls of the tumor is variable; their outer layer is generally constituted by skin, which is commonly very smooth, delicate, and thin, sometimes, however, retaining its normal thickness, or becoming red, rugose, and horny; their inner layers consist of the membranes of the spinal cord. In other, more rare cases, the skin is entirely wanting, and the walls are formed by the spinal membranes alone, which may be either in their natural condition, or may be red, swollen, and thickened. The contents of the tumor, consisting principally of the fluid described above, communicate with the subarachnoidean fluid contained in the vertebral canal. The swelling is usually formed of a single cyst, but there may be several, as in the multilocular variety of ovarian dropsy. Virchow, Gläser, and others describe, as congenital cystic hygroma of the sacral region, a tumor which sometimes attains a very large size, but is still in some cases curable by an operation; it is usually very complicated in its structure, and is accompanied by a cleft or incomplete development of the sacrum, but, in spite of it, does not generally communicate with the vertebral canal.

In very slight cases of hydrorachitis, the spinal marrow and the nerves originating from it are not all affected or changed. In many cases, however, the spinal marrow is flattened, and sometimes deviates from its accustomed route, being forced through the opening in the vertebræ, and partially contained in the swelling. The nerves are always more or less displaced; sometimes they are dragged out of the canal, and distributed over the inner surface of the cyst in a plexiform manner. The textural changes to which the spinal marrow is subject in hydrorachitis are various; it is often very much softened, or converted into a thin, diffluent mass; sometimes it has been found abnormally hard; sometimes it is not as large as in the natural state. These pathological changes are in most cases confined to those parts of the spinal cord which correspond with the seat of the cleft in the vertebral column. In some cases of hydrorachitis the spinal marrow may suffer more or less from arrest of development; instances are met with, though of rare occurrence, in which the central canal existing in the spinal marrow during fœtal life remains open, and is distended by serous effusion. In the most serious cases the spinal cord is in part, or in its entire length, wanting, either because it has not been developed at all, or because it has been destroyed or absorbed by the fluid collected in the subarachnoidean space; in the latter case, the contents of the sac contain occasionally fragments of the spinal marrow admixed with them.

Congenital hydrorachitis is, in the generality of cases, associated with congenital hydrocephalus; the latter affection being usually proportionate in degree to the former; in the highest forms of hydrorachitis, where the spinal marrow is completely absent, the brain is also generally wanting. Other congenital defects, with which hydrorachitis may be complicated, are hypospadias, extrophy of the bladder, imperforate anus, and hare-lip. Club-foot and similar deformities of the lower extremities are a very frequent complication. In some cases spina bifida is the only lesion, and the rest of the body is in a perfectly normal condition.

It would be interesting to know whether the cleft of the vertebral column is the primary, and the hydrorachitis the secondary affection, or whether the reverse is the case. The fact that a moderate deficiency in the vertebral rings, or a small cleft in the sacrum is sometimes found on dissection in subjects without any signs of hydrorachitis having ever existed, that the protrusion of the spinal envelopes does occasionally not take place until some time after birth, and that the tumor becomes larger and larger the longer it continues, seem to speak in favor of the first view; while the frequent coexistence of hydrocephalus with spina bifida, and the circumstance that the one will subside greatly while the other increases, and that plates of cartilage, constituting the laminæ, are not rarely found in the tissues covering the tumor, seem to indicate that the arrest of ossification is owing to the distention of the sac of the spinal column by the serous effusion. It is probable that in all cases, where the spinal marrow is destroyed, or has suffered important changes in its structure and course, a hydrorachitis was the primary affection; while in those cases where there is a protrusion of the spinal membranes alone, and the spinal marrow is in a normal condition, the lesion is secondary to the affection of the osseous parts.

The *symptoms* of hydrorachitis during life depend, principally, upon the situation and size of the tumor, the condition of the spinal marrow, and upon the complications which are present. The tumor, of which the situation, size, form, and external appearance have already been described, is either soft, flabby, and fluctuating, or it is full, hard, and shining. If it is of large size, it is often transparent. At its base, the bony margins of the cleft in the vertebral column can sometimes be distinctly felt. The swelling increases slightly during expiration, as well as crying and defecation in the vertical position, and diminishes during inspiration and rest; these symptoms, however, may be absent, if the communication with the spinal canal is very small. When pressed upon, the tumor gradually diminishes in volume, or completely recedes, but on removal of the force, the fluid reaccumulates, and the part regains its previous bulk. Pressure is painful when the external integuments or the inner wall of the tumor is inflamed, or when the tumor contains spinal marrow and nerves. In the latter case it may produce convulsions of the lower extremities. If the opening in the spinal canal is large, if hydrocephalus is present, and the fluid contained in the sac communicates freely with the cerebro-spinal fluid, pressure upon the tumor affects the fontanels, which are rendered prominent, and may produce general convulsions, symptoms of paralysis, and sopor. This effect of pressure will not, however, be noticed, if the neck of the sac is very small. In all cases where the disposition of the spinal marrow and its connection with the tumor is of a description that it may be easily irritated by pressure, by movements, and other external influences, general convulsions are apt to occur, and there may be contraction and rigidity of the lower extremities. When the pathological changes from which the spinal marrow has suffered are very considerable, or when there is great pressure upon it from fluid, the extremities may be paralyzed, cold, and emaciated, and there may be palsy of the sphincter muscles of the bladder and rectum. In most cases the general health of the children is seriously impaired; they become feeble, pale, and emaciated, vomit frequently, digest badly, are liable to suffer from inflammation of the urinary organs, and from ulceration of the skin and excoriation, and are disturbed in their sleep by pain. Some of the patients fall, a short time before death, into a state of stupor. Sometimes, however, if the disease is developed but to a moderate degree, it may remain without any influence upon the rest of the body.

Congenital hydrorachitis is nearly always *fatal*. In the highest degrees of the disease, when the cleft implicates the whole length of the vertebral column, and the spinal marrow and the brain are completely destroyed, or when there is destruction of a considerable part of the spinal marrow, the fœtus loses its vitality at an earlier or later period of intra-uterine life, and is prematurely expelled. If the child attains full development it may die during labor in consequence of sudden rupture of the sac, or because the large size of the tumor proves an impediment to the termination of labor. If the children are born alive, they may die sooner or later in consequence of the sudden escape of the contents of the tumor, the sac being opened either by ulceration, accident, or design; death, under these circumstances, is owing to convulsions from the pressure being taken off the brain in consequence of the loss of the cerebro-spinal fluid. In other cases the fatal termination is brought about by inflammation of the sac, which may extend to the membranes of the spinal cord, and even of the brain, or by convulsions. When the case is associated with hydrocephalus, paralysis of the inferior extremities, or involuntary discharge of the urine and feces, a speedy death is likely to ensue. Few children, affected with hydrorachitis, survive their birth longer than five or six months, while many perish in a much shorter time. It is true, life has sometimes been sustained until the age of puberty, and in one case, until the fifty-fifth year; but such instances are altogether of an exceptional character.

In the *treatment* of hydrorachitis, the use of internal remedies is of no avail, except to combat some of the intercurrent symptoms which may arise. How little is gained by external surgical treatment, experience has sufficiently shown. There is not the least prospect of success from surgical interference, whenever the disease is complicated with a high degree of hydrocephalus, or with other malformations of a serious character, when there is more than one tumor present, when the swelling has a broad base and is not covered by normal skin, when there are symptoms of the spinal marrow being much affected, or having prolapsed together with nerves, into the tumor. On the contrary, when the tumor has a narrow pedicle, is small, painless, completely covered with sound skin, when it seems to contain but little fluid, and is situated over the lower part of the vertebral column, there is a reasonable hope that a cure may occasionally be obtained.

Of the various modes of treatment devised for the cure of hydrorachitis, that by compression is perhaps one of the safest and best. It may be applied by means of a cup-shaped truss, lined with an air-cushion, so as to diffuse the pressure equally over the entire swelling; or the tumor may be painted over repeatedly with collodion, and the action of the latter aided by a common roller or adhesive straps. Several cases are reported in which a cure was obtained by a combination of methodic pressure with frequently repeated puncture; the latter is to be made subcutaneously with a very fine trocar or bistoury, the opening being well closed immediately afterwards, to prevent the introduction of air. Only a portion of the fluid should, however, be drawn off at a time, as a sudden evacuation of the tumor would be followed by the serious consequences already described. Sir Astley Cooper, early in life, treated successfully a case of cleft spine with compression alone, and, at a later period, succeeded in curing two cases by combining this method with repeated punctures, as proposed by Mr. Abernethy; in one of these cases the treatment was carried on during eighteen months, the tumor having been punctured thirty times before a cure was obtained.

The introduction of a seton, excision, or even simple incision of the tumor are nearly always followed by speedy death, and should never be attempted. The plan of tying the base of the tumor with a ligature, suggested by Benjamin Bell, with a view of removing the swelling and resisting the further propulsion of the spinal membranes, has been followed, in those cases in which it has been tried, by so little success, that there is no encouragement for its repetition. The patients died either from convulsions immediately after the operation, or a short time afterwards from an extension of the inflammation to the spinal cord and its membranes. The application of the ligature can be proper only in those very rare cases where the sac has a very narrow pedicle, and the aperture of communication is exceedingly small, but even under these circumstances the treatment by systematic compression, combined with repeated puncture, would be preferable, as it is connected with less risk to the patient, while offering at least equal chances of success. An instrument, resembling somewhat an enterotome, has been invented, in order to compress the base of the tumor, but has hardly any advantage over the ligature, upon the principle of which it acts. Excision of the tumor after ligation of the pedicle, and the application of the actual or potential cautery for the purpose of exciting adhesion between the opposing surfaces of the sac, are procedures which are generally followed by a fatal result, and are by no means to be recommended. The treatment of hydrorachitis by injection of iodine, as practiced in the two cases reported above, has been applied by different surgeons with various success. Professor Brainard of Chicago, by whom this treatment was first instituted, gives (*Chicago Medical Journal*, September, 1859, p. 517,) the result of ten cases treated in this manner; they occurred partly in his own practice, partly in that of Dr. Crawford, Chassaignac, Velpeau, and Nélaton. Of these ten cases, five, it is alleged, were cured, three died, and in two the results remained uncertain. The principal objection to this plan of treatment is, that it is impossible to circumscribe the resulting inflammation sufficiently to prevent it from spreading to the spinal marrow and its membranes.

In those cases of hydrorachitis, finally, in which surgical interference is deemed improper, the tumor should be protected, by a proper contrivance, from pressure, friction, and contact with the excretions; astringent lotions, fomentations, and ointments may be tried, with a view of producing thickening and shrinking of the walls of the sac, and tincture of iodine may be applied in order to promote absorption of the contents of the tumor.

